# The Implication of Physically Demanding and Hazardous Work on Retirement Timing

**DOI:** 10.3390/ijerph19138123

**Published:** 2022-07-01

**Authors:** Johanna Stengård, Marianna Virtanen, Constanze Leineweber, Hugo Westerlund, Hui-Xin Wang

**Affiliations:** 1 Stress Research Institute, Department of Psychology, Stockholm University, 11419 Stockholm, Sweden; constanze.leineweber@su.se (C.L.); hugo.westerlund@su.se (H.W.); huixin.wang@su.se (H.-X.W.); 2 School of Educational Sciences and Psychology, University of Eastern Finland, 80101 Joensuu, Finland; marianna.virtanen@uef.fi; 3 Division of Insurance Medicine, Department of Clinical Neuroscience, Karolinska Institutet, 17177 Stockholm, Sweden

**Keywords:** physical job demands, physically demanding work tasks, physically hazardous work environment, retirement timing, actual retirement, age interactions

## Abstract

The need to retain individuals longer in the workforce is acknowledged in many high-income countries. The present study therefore aimed to examine the importance of physically demanding work tasks (PDWT) and physically hazardous work environment (PHWE) in relation to retirement timing among pensionable workers (≥61 years). A particular question was whether PDWT and PHWE increased in importance with age. Six waves (2008–2018) of the Swedish Longitudinal Occupational Survey of Health (SLOSH) were used (*n* = 5201; 56% women and 44% men; mean age at first survey was 61.0 (SD 2.0) years). Discrete time-event history analysis, stratified by socioeconomic position and gender, showed that among blue-collar workers, PDWT and PHWE were associated with an increased likelihood of retiring within the next two years. With increasing age, high-level PHWE was associated with higher probability of retiring among blue-collar men, whereas heavy PDWT was associated with lower probability of retiring among blue-collar women. Among white-collar workers, having at least some PDWT compared to no PDWT was associated with a lower likelihood of retiring within the next two years. With increasing age, exposure to PHWE was associated with higher probability of retiring among white-collar women. These results suggest that to delay retirements, organizations could offer their older employees, especially blue-collar workers and the oldest white-collar women, alternatives to PDWT and PHWE.

## 1. Introduction

The necessity to retain individuals longer in the workforce due to ageing populations is acknowledged in many high-income countries [1,2]. However, less than two percent of the European population aged 65–69 worked in 2018 [3]. People decide to retire for a variety of reasons, including their economic and family situation, such as the need to care for a relative, but also depending on the psychosocial working conditions at work. In order to prolong working life, development of sustainable working conditions in general and for older workers in particular may be needed by organizations. For instance, a study on Swedish healthcare workers aged 55–64 years demonstrated associations between good physical and psychosocial working conditions and perceptions of being able to work until or beyond 65 years, which is the normative retirement age in Sweden [4]. Although there are studies on the impact of physical demands on disability pension [5,6,7], the importance of physical working conditions on the timing of old-age retirement is still scarce. In particular, whether these conditions become more significant as one approaches and passes the normative retirement age has not been investigated.

Physical job demands can be defined as aspects of the job that require sustained physical effort or that constitute an unfavorable physical working environment and are thus associated with physiological and/or psychological individual costs according to the job Demands–Resources (JD–R) theory [8,9]. Besides physically demanding work tasks (PDWT) that relate to body movements, such as lifting heavy equipment or working in awkward positions, individuals could be affected by potentially physically hazardous work environment (PHWE), such as noise, toxins, etc.

Of the studies focusing on working conditions and non-disability early retirement, a systematic review [10] found only three studies on physical job demands appropriate for inclusion and among these, only one supported an association between higher PDWT and early retirement. A more recent systematic review on working conditions and retirement timing—without a specific focus on early retirement—stated that they “did not include physical working conditions as they are often not measured, or not well measured, in research studies on ageing” [11] (p. 1). Recently, only a limited number of studies have further explored the relationship between physical working conditions and working after statutory/normative retirement age, showing mixed results. Three studies from the Nordic countries [12,13,14] found support for an association between low PDWT and working beyond the state pension age, whereas a Dutch study did not support such an association [15]. However, the former three measured PDWT only with a single item. Another limitation is that in one of these studies [13], PDWT was retrospectively evaluated and in others, the exposures were measured at different lengths of time before retirement within a single study cohort; for example, up to five years before [15] and as early as at the age of 55–59 years [12]. To evaluate the impact of working conditions several years before retirement age does not per se reveal the impact of physical working conditions close to retirement on the decision (or need) to retire. The worker’s working environment may have changed substantially during this time period, for instance through job changes, reorganizations and job crafting (i.e., proactively adjusting one’s own job conditions). A few studies have examined and reported an association between PHWE and actual old-age retirement timing [16]. However, studies investigating associations between physical demands and old-age retirement typically do not separate blue- from white-collar workers. As a consequence, the results may simply reflect a systematic difference in retirement age norms between blue- and white-collar occupations. Therefore, more research on the impact of both PDWT and PHWE on old-age retirement, separating white- and blue-collar workers is warranted.

According to the JD–R theory, there may be (health) costs associated with both PDWT and PHWE [17]. At the same time, individuals strive to increase or protect their resources (such as, energy, time and health) according to the conservation of resources (CoR) theory [18]. Thus, a person who has reached retirement age may choose to retire if job resources cannot compensate for energy and time loss and/or deterioration of health or in order to gain resources associated with retirement, such as more time to spend on sleeping mornings or to be with grandchildren. Thus, it is reasonable to expect that physical work environment may act as a push (risk) factor towards retirement [19].

Moreover, it is conceivable that physical demands, with increasing age, play an even greater role for retirement timing as it may be harder to keep up with high physical demands when aging. For example, one study on workers aged 18 to 59 years found a tendency for a stronger association between PDWT and lower self-rated health among older compared to younger workers [17]. Another study, including only men aged 62 years and older, reported that PDWT was more strongly associated with retirement for the oldest age groups [20]. If age moderates the effect of PDWT also in women is to the best of our knowledge not known. Likewise, whether the effect of PHWE varies by age has not been thoroughly studied yet. With regard to job resources, similar age differences have been shown. For example, one recent study found support for a (linear) age effect on the association between psychosocial resources and retirement timing among workers who had reached pensionable age, such that more resources played a stronger role for continuing working among the oldest [21].

Previous research shows that socioeconomic position is associated with timing of labor market exits, especially with regard to health-related exits [22]. Lower compared to higher education [22,23,24] and blue-collar compared to white-collar jobs [22] have been found to be associated with “all-sorts” of exits. One Finnish study on older workers found that upper non-manual employees and individuals with higher educational levels had higher likelihood of retiring later [25]. Another Finnish study found that white-collar workers were two times more likely to work beyond their individual pensionable age than blue-collar workers [14]. Since physical working conditions also differ significantly between occupations with generally more demanding conditions in jobs in low socioeconomic positions (blue-collar work) [14], it is important to study blue- and white-collar workers separately.

Although the Swedish pension system is flexible, 65 years is still considered the normative retirement age [26,27]. In fact, in 2018, the average age for exit from the labor market in Sweden was 63.6 for women and 64.6 for men [27], whereas the proportion of those aged 65–74 who were still in paid work at least one hour per week was 21% among men and 13% among women [28]. The Swedish labor market is highly gender-segregated—with 70% of women working in female-dominated occupations and 66% of men working in male-dominated occupations [29]. Thus, also men and women should be studied separately in regard to retirement timing.

Taken together, we expect that both PDWT and PHWE increase the likelihood of retirement for workers of pensionable ages. Specifically, we hypothesize that:

1. Higher PDWT increases the likelihood of retirement (within two years).
2. Exposure to PHWE increases the likelihood of retirement (within two years).
3. The influence of PDWT on retirement vs. continued work increases with age.
4. The influence of PHWE on retirement vs. continued work increases with age.

We will stratify all our analyses by socioeconomic position (i.e., blue- or white-collar work) and by gender.

## 2. Materials and Methods

### 2.1. Sample and Procedure

The Swedish Longitudinal Occupational Survey of Health (SLOSH) is a nationwide prospective cohort study, collected biennially since 2006 through postal questionnaires. The SLOSH cohort constitutes an approximately representative sample of the Swedish working population as it follows up the participants of the biennial Swedish Working Environment Surveys (SWES), which invited a subsample of the workers (16–64 years) in the Labour Force Survey (LFS), which consists of a randomly sample of the entire Swedish population draw every second years. Both the SWES and the LFS are conducted by Statistics Sweden (SCB). When SLOSH started in 2006, the SWES 2003 respondents were invited to take part in the first SLOSH data collection. Over time, more SWES populations have been included, and in 2018, SLOSH comprised the SWES respondents from 2003–2011, in total over 40,000 individuals. There are two versions of the SLOSH questionnaire: one for those currently working (at least 30% of a full-time job) and the other for those not working or working <30% of a full-time job. SLOSH covers a broad range of questions about work organization and work environment, and health measures. Additionally, there are links to national register data on health and demographic variables. Details on the SLOSH collections have been described elsewhere [30]. The SLOSH and the present study have been approved by the Regional Research Ethics Board in Stockholm.

#### Inclusion Criteria

The present study comprised participants from SLOSH 2008–2018. At the time of the data collections of the present study, it was possible to retire (part- or full time) from the age of 61 with collected-earnings-related state pension. A supplementary pension for those who have had little or no pensionable income during their lives was paid from the age of 65. Until their 67th birthdays, employees had a legal right to retain their employment, and thereafter the person could continue to work in agreement with the employer (https://www.pensionsmyndigheten.se acceseed on 31 May 2022).

Employees entered the study population at the wave at which they reached the age of at least 59 (i.e., reaching the lowest state pension age 61 years at the follow-up wave) (*n* = 7548; 2008–2016). In the present study, an observation consists of a pair of waves, i.e., “baseline wave” (any single wave) and “follow-up wave” (the subsequent wave). (Consequently, those who became eligible first in 2018 were excluded.) Observations were included if the individual at baseline answered the questionnaire for those in work and at the follow-up answered either the questionnaire for those in work or, in the case not being in paid work, the questionnaire for non-working in which they indicated being full-time retired with old-age pension and/or disability pension. Consequently, an individual could contribute to up to five observation pairs. Waves where a person indicated that he/she worked less than 30% were not considered. The final dataset (5201 individuals; 8791 observations) comprised observations from those individuals who had completed at least one “work to work” or “work to retirement” transition between two subsequent waves. Of those workers who had a transition to retirement (*n* = 2806), very few (*n* = 40) stated the reason was disability retirement. Those were kept in the final dataset. After retiring, a few persons got re-employed (*n* = 26), these individuals only contributed to the analyses with their first “work to retirement” transition and any previous “work to work” transition.

### 2.2. Measures

#### 2.2.1. Outcome Variable

Retiring: retired within (1) vs. still in work after (0) two years (measured at the follow-up). Individuals who answered the questionnaire for those in work (i.e., working at least 30% of a full-time job) were considered still working and individuals who answered the questionnaire for non-working and stated being full-time retired with old-age pension and/or disability pension were considered retired.

#### 2.2.2. Stratification Variables

Socioeconomic position was provided by Statistics Sweden based on the Swedish socio-economic classification (SEI) [31], which is based on the participants’ survey responses about employment (“What is/was your main occupation/profession?”) provided at each survey. We excluded those being self-employed. All other were divided into two categories (1) Blue-collar workers (*n* = 2789 observations: unskilled and semi-skilled manual workers (*n* = 1355 observations; 48.6%) and skilled manual workers (*n* = 1434 observations, 51.4%)) and (2) white-collar workers (*n* = 6002 observations: assistant non-manual employees (*n* = 1262 observations; 21.0%), intermediate non-manual (*n* = 2898 observations; 48.3%), and professionals and other higher non-manual employees (*n* = 1842 observations; 30.7%)). Information on gender was register-based.

#### 2.2.3. Exposure Variables

Two different types of physical demands were assessed on two scales: physically demanding work tasks (PDWT) related to body movements, and physically hazardous work environment (PHWE) related to the person’s immediate work surroundings. Response alternatives were (1) “not at all”, (2) “a little, perhaps 1/10 of the time”, (3) “about ¼ of the time”, (4) “about half of the time”, (5) “about ¾ of the time”, or (6) “almost all the time”. *PDWT* was measured with a three-item scale (physical labor, heavy lifting, and awkward working positions) [32]. A sum-index was estimated (value range 3–18) with Cronbach’s alpha = 0.84 for blue-collar workers and Cronbach’s alpha = 0.78 for white-collar workers. *PHWE* was measured with six questions concerning whether the person was exposed to any of the following at work: (a) noise, (b) poor or excessively bright light, (c) excessive heat, cold or draught, (d) vibrations that make your whole body shake and vibrate, (e) toxins or irritants, and (f) tangible risk of injury. Similar questions are used by Statistics Sweden in their biennially surveys of the working environment in Sweden. Since the items refer to different aspects of the physical work environment not necessarily related, the response alternatives were first dichotomized (0: not (or very little) exposed (response alternatives 1–2) vs. 1: exposed (response alternatives 3–6)) and thereafter a sum-index of the number of indicators exposed to was estimated for PHWE (value range 0–6 indicators). Because the distributions of PDWT and PHWE differed substantially between blue- and white-collar workers and the scales were strongly positively skewed—especially for white-collar workers since a large portion of them reported few or no physical demands—it was not meaningful to categorize work demands in the same way in the stratified analyses. For blue-collar workers, PDWT and PHWE measures were divided into three approximately equal-sized categories: 0 “light PDWT” (37.0%; value range 3–7), 1 “moderate PDWT” (29.4%; value range 8–11), or 2 “heavy PDWT” (33.6%; value range 12–18) and 0 “low-level PHWE” (27.7%; 0 indicators), 1 “moderate-level PHWE” (43.4%; 1–2 indicators), or 2 “high-level PHWE” (28.9%; 3–6 indicators). For white-collar workers, PDWT and PHWE measures were dichotomized into binary indicators with the cut-off representing their median value: 0 “without PDWT” (57.4%; value 3) or 1 “with PDWT” (42.6%; value range 4–18) and 0 “unexposed to PHWE” (57.1%; 0 indicators) or 1 “exposed to PHWE” (42.9%; 1–6 indicators).

### 2.3. Statistical Analysis

We performed discrete-time event history analysis using a 2-level structure with the observations (transitions) at different ages nested within individuals and retirement at follow-up as a binary outcome variable (Stata version 17.0). With six waves, the selected sample admits up to five “pairs of waves” (observations), between two successive waves (“baseline” and “follow-up” waves). Two types of transitions were specified: from work to work and from work to retirement. Age served as the timing of the event and was based on register data corresponding to the respondent’s age at the particular baseline wave of that particular observation pair. Because the age–retirement distribution (i.e., odds ratios of retirement timing as a function of age) in our sample was approximately inverted-U-shaped, we included both a linear and a quadratic age term in all models. Note that 58 was first subtracted from age, so that value 1 corresponded to the lowest possible age of 59 years.

In Model 1 (minimally adjusted), linear and quadratic age terms and wave (categorical) were included as covariates. Model 2 and onwards were additionally adjusted with education, working time (working full- vs. part-time), marital status (married/cohabitant vs. single), and parental status (children living at home vs. not), and caring for a relative (yes vs. no), also referred to as fully adjusted. First, in Model 1–2 (minimally and fully adjusted), PDWT and PHWE were included by sum-scale index. For the remaining models (Model 3–4; all fully adjusted), the analyses were stratified by socioeconomic position (blue-collar or white-collar workers) and gender, and PDWT and PHWE were represented by three-level (categorical) variables for blue-collar workers and binary variables for white-collar workers. The Spearman’s correlation between PDWT and PHWE was 0.33, *p* < 0.001 (*n* = 2688 observations) for blue-collar workers and 0.31, *p* < 0.001 (*n* = 5881 observations) for white-collar workers.

#### 2.3.1. Age Moderating Effects

Whether the associations between physical demands and retirement timing differed in strength with age was tested by including an interaction term between linear age and PDWT or PHWE on retirement timing (Model 4) to Model 3. Since there were too few observations for the older age groups to make predictions for any linear relationship with age, this final test was limited to age <68.

#### 2.3.2. Sensitivity Analyses

The following sensitivity analyses were performed: (1) an interaction term between binary age (cut-off 59–63 years vs. 64–76 years at baseline) and PDWT or PHWE was included instead of a linear interaction term. In this analysis, the older age category included observations where the individual at the follow-up was at least 65 years old, which means that they had reached the Swedish normative retirement age, (2) transitions to disability retirement were excluded, (3) a limit for the baseline age was set to <70 years, (4) self-rated health and physical and psychological work ability were entered as covariates.

## 3. Results

At the initial baseline survey (time point varies between the participants), there were 56% (*n* = 2934) women and 44% (*n* = 2267) men. In total, 33% were blue-collar workers and 67% white-collar workers. Moreover, 75% worked full-time and 25% worked part-time, 79% were married/cohabiting, 11% had children living at home, and 16% were caring for a relative. In total 14%, had elementary school education, 41% secondary school education, 5% short higher education, and 40% university education. Finally, the mean age at the initial survey was 61.0 (SD 2.0) years, ranging from 59 to 71 years.

The analyses including all individuals, minimally adjusted for age (linear and quadratic terms) and wave (categorical) (Model 1) showed that there were increased odds of retiring two years later if having higher PDWT or PHWE; that is, one-unit increase in the PDWT sum-scale resulted in a greater odds (OR = 1.02, 95%CI 1.00–1.03 (*n* = 5183 individuals; 8726 observations) and in the PHWE scale OR = 1.06, 95%CI 1.01–1.10 (*n* = 5155; 8634 observations). However, the associations were no longer statistically significant in the fully adjusted Model 2: for PDWT OR = 1.00, 95%CI 0.98–1.01 (*n* = 5012; 8243 observations) and for PHWE OR = 1.04, 95%CI 0.99–1.08 (*n* = 4983; 8176 observations).

The fully adjusted analyses stratified by socioeconomic position and gender (Model 3) showed that for blue-collar workers (here PDWT and PHWE are represented by three-level variables) there was greater odds of being retired two years later if having heavy compared to light PDWT (Table 1). When stratified further by gender, this association remained statistically significant both for women and men. With regard to PHWE, blue-collar workers with high-level compared to low-level PHWE had higher odds of retiring. This association was significant among women whereas among men the association only reached the borderline significance level. Regardless of gender, blue-collar workers with moderate-levels of PDWT or PHWE had not significantly higher odds of retiring compared to workers with low-levels of PDWT or PHWE.

White-collar workers with PDWT (compared to without) had lower odds of retiring within the next two years suggesting that workers with PDWT may continue their work to a greater extent than those without PDWT (Table 2). This applied for both women (borderline significant) and men. Exposure to PHWE did not increase the odds of retiring within the next two years neither for white-collar women nor men.

### 3.1. The Moderating Effect of Age for Blue-Collar Wokers (Model 4)

With regard to a possible moderating effect of age (limited to observations where individuals were <68 years) on the relationship between PDWT or PHWE and retirement timing: for blue-collar women a linear moderating effect of age (Model 4) was found for heavy compared to light PDWT, such that with increasing age heavy PDWT decreased rather than increased the likelihood of retirement (Table 3).

**Figure 1:**
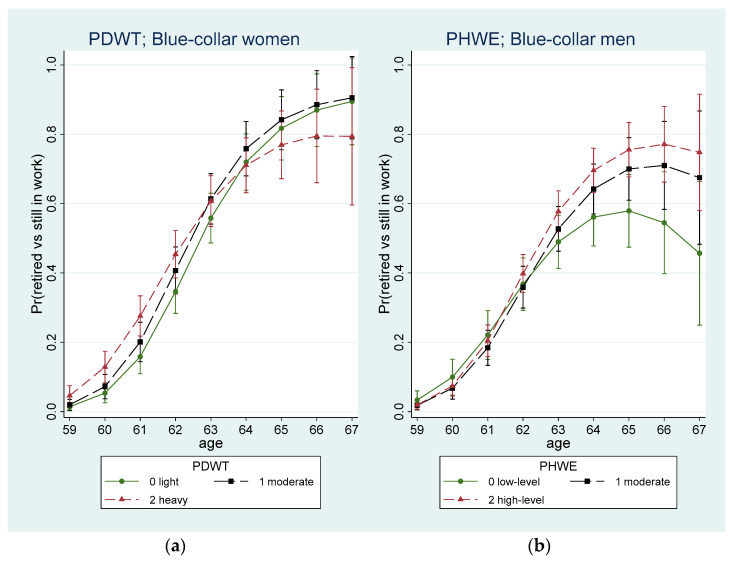
Predictive marginal probability (and 95% CI) of retiring (vs. still in work) at 2-year follow-up by age and (**a**) PDWT for all blue-collar women; (**b**) PHWE for blue-collar men.

Figure 1a presents the adjusted predictive marginal probability of retiring (vs. still in work) at 2-year follow-up by baseline age and PDWT status, indicating that the probability for retiring was higher for blue-collar women with heavy compared to light PDWT up to the age of 63 and thereafter the relationship changed direction so that individuals with heavy compared to light PDWT had lower probability of retiring. For blue-collar men, a linear moderating effect of age was found such that with age there was a significant increase in the likelihood of retirement for those with high-level PHWE and borderline significant for those with moderate PHWE compared to those with low-level PHWE (Table 3; Figure 1b).

### 3.2. The Moderating Effect of Age for White-Collar Wokers (Model 4)

**Figure 2:**
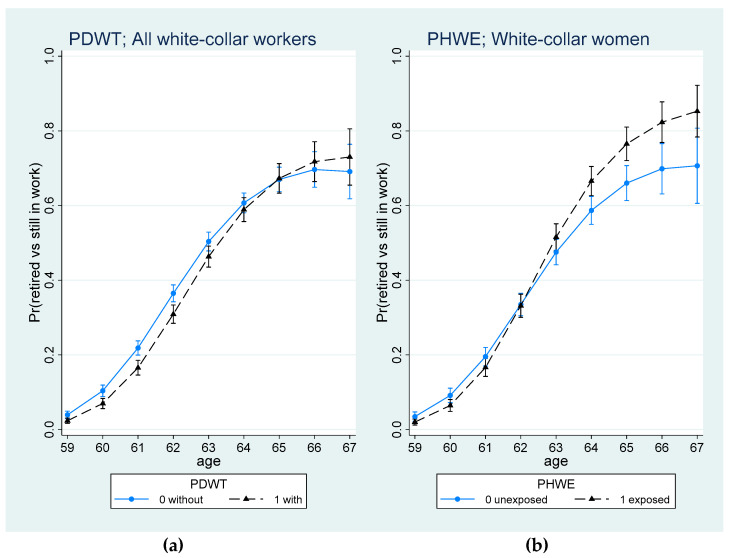
Predictive marginal probability (and 95% CI) of retiring (vs. still in work) at 2-year follow-up by age and (**a**) PDWT for all white-collar workers; (**b**) PHWE for white-collar women.

For white-collar workers (Table 4), a significant linear moderating effect of age for the association between PDWT and retiring (within two years) was found, such that with age, exposure to PDWT increased the likelihood of retirement. Figure 2a presents the average marginal effects of (with) PDWT (vs. without PDWT) on retiring for different ages, indicating that the relationship between PDWT and retiring for younger ages was negative up to the age of 65, but became positive after 65 years. For analyses stratified by gender, the interaction term was non-significant.

A significant interaction term between PHWE and age indicated that with age, the odds of retiring within two years increased if white-collar workers were exposed to PHWE (Table 4). However, the interaction term was only significant for women, but not for men. Results of the average marginal effects of being exposed to PHWE (vs. unexposed) on retiring for white-collar women at different ages (Figure 2b) indicate that the influence of PHWE on retirement increased with age (at least up to 67).

### 3.3. Sensitivity Analyses

Sensitivity analyses where the linear age interaction term was replaced by an interaction term between PHWE or PDWT and binary age (cut-off value 64; also including observations with ages 68 and above) (Supplementary Tables S1 and S2), similar trends and directions of the relationships were found. Neither did the exclusion of the limited number of transitions between work and disability retirement change any conclusions. To be noted for blue-collar men the influence of high-level PHWE on retirement turned significant from borderline significant, whereas the influence of linear age on the same association turned borderline significant from significant (Supplementary Table S3). Setting an age-limit to <70 years in Model 3 did not change any conclusions. Finally, when self-rated health and physical and psychological work abilities were included in the models, the results were only mildly affected (Supplementary Tables S4 and S5). To be noted for blue-collar men, the influence of PHWE on retirement timing changed to be non-significant from borderline significant.

## 4. Discussion

The aim of the present study was to examine the importance of physically demanding work tasks and physically hazardous work environment—PDWT and PHWE—in relation to retirement timing among pensionable workers. Because retirement timing [33] and physical working conditions differ between occupations, with generally more demanding conditions in lower status jobs [14], and because the Swedish labor market is highly gender-segregated [29], we stratified the analyses by both socioeconomic position and gender. Another aim was to examine whether the influence of PDWT and PHWE on retirement timing was dependent on age. Models took into account age (linear and quadratic term), wave, education, working time, marital and parental status, and caring for a relative.

For blue-collar workers, heavy PDWT or high-level PHWE increased the likelihood of retiring within the next two years. With increasing age, high-level (and moderate-level) PHWE was associated with a higher likelihood of retirement among blue-collar men, whereas heavy PDWT rather was associated with a lower likelihood among older blue-collar women. With regard to white-collar workers, unexpectedly, those without any PDWT compared to those with PDWT had a higher likelihood of retiring within the next two years. However, there was a small tendency that with increasing age, having at least some PDWT increased the probability of retirement. Additionally, the influence of PHWE increased with increasing age, but only for white-collar women.

### 4.1. Physically Demanding Work Tasks

Our finding that blue-collar workers with heavy PDWT compared to those having light PDWT had higher probability of retiring within the next two years is in line with some previous studies [12,13] and supporting our hypothesis in light of both JD–R and CoR theories [9,18]. Our finding did not differ between women and men, that is, independent of gender, blue-collar workers with heavy PDWT had a higher probability of retiring. However, we did not find support for that PDWT became more important for blue-collar men or blue-collar women when ageing, where for the latter group the tendency rather was in the opposite direction (after age 64), i.e., with age, heavy compared to light PDWT was associated with a lower likelihood of retiring within the next two years among blue-collar women. This was a bit surprising as we expected that heavy PDWT would become more burdensome due to decreased physical work ability when ageing—which would affect health and increase the need or incentives to retire—and one previous study supported such tendencies among working men, however in a study sample including both blue- and white-collar men [20]. One interpretation of our finding may be that such a process is already at play around the age of 60 and does not change much subsequently. Plausibly, mainly the healthiest individuals, who can handle heavy physically demanding tasks, remain in the labor market (the healthy worker effect) or that older workers exchange their physically demanding tasks for less demanding ones [34] either with the same or a different employer [20]. Another plausible explanation for the unexpected finding—that with age (after about 64) blue-collar women had a decreased likelihood of retiring if having heavy PDWT—may be that some workers in female-dominated occupations characterized with heavy PDWT, e.g., preschool staff with short education and assistant nurses, are less economically compensated [35,36] and therefore cannot afford to retire. This needs to be further investigated.

Unexpectedly, both for white-collar women and men, there was a tendency that having at least some PDWT compared to having no PDWT was associated with a lower probability of retiring within the next two years. However, generally, the PDWT levels were low among white-collar workers and therefore may not be so exhausting after all. On the contrary, these low levels of PDWT may be perceived as rather beneficial, perhaps motivating individuals to continue working yet another year, since too much sedentary desk work has proven to be unhealthy [37]. Another possibility, although we adjusted for controlled educational level, is that level of PDWT reflects a status-difference within white-collar occupations [38], such that, occupations with no (or a few) PDWT have higher salaries and better benefits allowing to retire earlier. However, for white-collar workers, our findings suggest that with age (up to 67 years) there may in fact be a small increase in the influence of PDWT on retirement. One possible explanation may be that even few PDWT eventually become more burdensome with age [17,20], and thus after a certain age (after 65 according to our results) PDWT increases the likelihood of retirement. However, in analyses stratified by gender, this relationship was not significant anymore.

Based on our slightly different findings for different socioeconomic position, one reason some previous studies did not support a relationship between PDWT and retirement timing, e.g., [15] could be that they did not separate blue- from white-collar workers. In fact, for instance, Virtanen and colleagues [14] found that PDWT mediated the link between socioeconomic position and retirement timing.

### 4.2. Physically Hazardous Work Environment

For blue-collar workers, we found support for an association of high-level PHWE with higher probability of retiring among blue-collar women and a tendency among blue-collar men, which is in line with the findings of a previous Finnish study [16]. However, Böckerman and Ilmakunnas [16] did not separate blue- from white-collar workers. A positive influence of linear age on the relationship between PHWE and retirement was found for blue-collar men only, meaning that high-level compared to low-level PHWE became more important for retirement when getting older. Similarly, there was a tendency that moderate levels of PHWE compared to low-level PHWE increased in importance with age. Thus, although the overall influence of PHWE on the likelihood of retiring was more pronounced for blue-collar women, with age the influence grew in importance only for blue-collar men.

Among white-collar workers, we did not find support for associations between exposure to PHWE and the likelihood of retiring in general or when stratified by gender. However, our results suggest that the importance of PHWE for retiring within the next two years increased with age, such that there was a linear trend where PHWE grew in importance annually (up to 67 years). In analyses stratified by gender, this relationship was still identified among white-collar women whereas not among white-collar men. Women predominantly work in occupations with many human contacts, such as health care, social care and schools, which are known to be stressful. For example, noise is common both in schools [39] and in intensive care [40], which has been found to be related to physical exertion among nurses [41]. It is reasonable to assume that PHWE may have stronger negative health effects with age, either through a cumulative effect over time and/or due to reduced physiological functioning [17], and thus with age become a potential reason for retiring.

### 4.3. Strengths and Limitations

Strengths of the present study include the use of a sizeable, approximately representative prospective cohort of the Swedish workforce with several measurement points. We also stratified the analyses by socioeconomic positions and gender, which we consider being an important strength of the study since the results otherwise simply might have had reflected differences in retirement timing between white and blue-collar workers. Moreover, as the working conditions in terms of physical demands for white and blue-collar workers diverge substantially, we operationalized their levels of PDWT and PHWE differently (three-category variables for blue-collar workers and binary variables for white-collar workers because very few of them had PDWT or PHWE). This means that we cannot compare the effects of certain levels of PDWT/PHWE between blue- and white-collar workers. The findings for blue-collar workers showed a more expected pattern—where higher values of PDWT/PHWE were associated with a higher probability of retirement within the next two years—whereas results for white-collar workers in several cases were contrary to expectations. The reason could be that for white-collar workers, only a small amount of exposure was sufficient to be categorized into having PDWT or being exposed to PHWE and this small amount was not enough to influence retirement decision. For example, only one indicator of an environmental hazard, e.g., noisy or poor light, about 25% of the time exposed was enough to categorize a white-collar worker as exposed. Instead, perhaps other forces are in action, for instance, different occupations with different retirement timing norms. In future research on physical demands and retirement timing, we suggest to especially target blue-collar workers, where a finer-grained categorization could be utilized cf [14,33].

Second, although, the baseline age for each transition was known—the time point when physical job demands were measured—the exact dates of retirement are unknown for those who reported that they were retired two years later. Possibly this may have underestimated any age influence on the associations between physical demands and retirement timing. It is possible that more frequent data collections or register data on annual sources of income (pension vs. labor) [42] would have shown stronger associations. Third, observations where the persons worked less than 30% of a full-time job were not considered, and thus any influence of physical job demands for reducing work hours from ≥30% to <30%, or from <30% to full retirement could not be revealed. Future research could focus on the extent to which physical demands lead to individuals reducing their working hours.

Finally, we did not control for work ability or self-rated health in our analyses, since they may act as mediators in the relationship between physical work demands and retirement timing [43,44], and thus, in fact true associations may be underestimated if including those as covariates. In sensitivity analyses where these variables were included, the influence of PHWE (but not PDWT) on the likelihood of retiring decreased somewhat among blue-collar workers, suggesting that future studies could test health as a mediator in the association. That controlling for health did not affect the association between PDWT and retiring, and only mildly influenced the association between PHWE and retirement timing, probably is partly due to the healthy worker effect, that is less healthy individuals tend to drop out of studies or they may have left earlier on with a disability pension.

## 5. Conclusions

As a consequence of ageing populations, people in high-income societies need to prolong their working lives and this study suggests that physical demands of work may be significant. The present study also suggests that when approaching their retirement age, both blue-collar men and women with heavy physically demanding work tasks or in a high-level physically hazardous work environment have an increased probability of retirement within the next two years compared to blue-collar workers with better working conditions. In order to prolong employees’ working life, organizations should therefore make an effort to improve the physical working environment, reduce hazardous element to a minimum and enable workers in pensionable age to change heavy work tasks for lighter ones. For blue-collar men and white-collar women especially, exposure to a physically hazardous work environment seem to become more important with age as a factor that increases the likelihood of retirement. Consequently, it may be necessary to investigate whether there are certain occupations in which it is difficult to work at older ages and where there is a need to tailor interventions to reduce hazardous aspects of the job.

## Figures and Tables

**Table 1:** Blue-collar workers. Odds ratio (OR) and 95% confidence interval (CI) of retired (1) vs. still in work (0) two years later in relation to PDWT and PHWE in trichotomised variables. Separate models.

	N_obs_ (N_ind_)	All Blue-Collar Workers OR (95% CI)	N_obs_ (N_women_)	Women OR (95% CI)	N_obs_ (N_men_)	Men OR (95% CI)
**PDWT**	2543 (1649)		1204 (796)		1339 (853)	
light (ref)	939	–	435	–	504	–
moderate	763	1.15 (0.90; 1.48)	372	1.24 (0.86; 1.78)	391	1.04 (0.74; 1.47)
heavy	841	1.45 (1.14; 1.85) **	397	1.53 (1.07; 2.20) *	444	1.42 (1.02; 1.98) *
**PHWE**	2518 (1631)		1191 (787)		1327 (844)	
low-level (ref)	686	–	414	–	272	–
moderate	1098	1.12 (0.88; 1.43)	642	1.08 (0.78; 1.49)	456	1.20 (0.82; 1.76)
high-level	734	1.41 (1.07; 1.86) *	135	2.01 (1.19; 3.41) *	599	1.42 (0.98; 2.06) ^†^

Fully adjusted for age (linear and quadratic), wave (categorical), education, marital status, parental status, working time, and caring for a relative (Model 3). ** for *p* < 0.01; * for *p* < 0.05; ^†^ for 0.05 ≤ *p* < 0.10; N_obs_ = number of observations; N_ind_ = number of individuals.

**Table 2:** White-collar workers. Odds ratio (OR) and 95% confidence interval (CI) of retired (1) vs. still in work (0) two years later in relation to PDWT and PHWE in dichotomised variables. Separate models.

	N_obs_ (N_ind_)	All White-Collar Workers OR (95% CI)	N_obs_ (N_women_)	Women OR (95% CI)	N_obs_ (N_men_)	Men OR (95% CI)
PDWT	5700 (3448)	0.82 (0.72; 0.94) **	3443 (2057)	0.84 (0.70; 1.00) ^†^	2257 (1391)	0.78 (0.63; 0.96) *
PHWE	5658 (3437)	1.09 (0.95; 1.24)	3411 (2048)	1.10 (0.92; 1.31)	2247 (1389)	1.04 (0.84; 1.30)

Fully adjusted for age (linear and quadratic), wave (categorical), education, marital status, parental status, working time, and caring for a relative (Model 3). ** for *p* < 0.01; * for *p* < 0.05; ^†^ for 0.05 ≤ *p* < 0.10.

**Table 3:** Blue-collar workers. Moderating effect of linear age (limited to <68 years at baseline wave) on the association between PDWT or PHWE and retired (vs. still in work) two years later (age * PDWT/PHWE). Odds ratio (OR) and 95% confidence interval (CI) presented. Separate models for PDWT and PHWE.

	All Blue-Collar Workers	Women	Men
	OR (95% CI)	OR (95% CI)	OR (95% CI)
**PDWT**			
Main effect: light (ref)			
	–	–	–
moderate	0.97 (0.45; 2.09)	1.47 (0.43; 5.03)	0.78 (0.29; 2.13)
heavy	1.70 (0.83; 3.49)	4.40 (1.47; 13.22) **	0.91 (0.34; 2.45)
Interaction with linear age: age#light (ref)			
	–	–	–
moderate	1.04 (0.89; 1.22)	0.97 (0.74; 1.28)	1.06 (0.87; 1.30)
heavy	0.96 (0.83; 1.12)	0.77 (0.61; 0.99) *	1.10 (0.90; 1.36)
**PHWE**			
Main effect: low-level (ref)			
	–	–	–
moderate	0.65 (0.31; 1.36)	0.98 (0.35; 2.74)	0.44 (0.15; 1.33)
high-level	0.94 (0.43; 2.06)	2.74 (0.70; 10.67)	0.45 (0.16; 1.28)
Interaction with linear age: age#low-level (ref)			
	–	–	–
moderate	1.12 (0.96; 1.31)	1.02 (0.81; 1.28)	1.21 (0.98; 1.51) ^†^
high-level	1.09 (0.92; 1.28)	0.91 (0.66; 1.26)	1.26 (1.02; 1.56) *

Fully adjusted for age (linear and quadratic), wave (categorical), education, marital status, parental status, working time, and caring for a relative (Model 4). ** for *p* < 0.01; * for *p* < 0.05; ^†^ for 0.05 ≤ *p* < 0.10.

**Table 4:** White-collar workers. Moderating effect of linear age (limited to <68 years at baseline wave) on the association between PDWT or PHWE and retired (vs. still in work) two years later (age * PDWT/PHWE). Odds ratio (OR) and 95% confidence interval (CI) presented. Separate models for PDWT and PHWE.

	All White-Collar Workers	Women	Men
	OR (95% CI)	OR (95% CI)	OR (95% CI)
**PDWT**			
main effect	0.53 (0.36; 0.79) **	0.57 (0.34; 0.97) *	0.49 (0.27; 0.89) *
interaction with linear age	1.10 (1.01; 1.19) *	1.08 (0.97; 1.21)	1.10 (0.98; 1.24)
**PHWE**			
main effect	0.63 (0.43; 0.93) *	0.47 (0.28; 0.79) **	1.12 (0.62; 2.04)
interaction with linear age	1.13 (1.04; 1.22) **	1.20 (1.08; 1.34) ***	0.99 (0.87; 1.11)

Fully adjusted for age (linear and quadratic), wave (categorical), education, marital status, parental status, working time, and caring for a relative (Model 4). *** for *p* < 0.001; ** for *p* < 0.01; * for *p* < 0.05.

## Data Availability

Given restrictions from the ethical review board and considering that sensitive personal data are handled, it is not possible to make the data freely available. Access to the data may be provided to other researchers in line with Swedish law and after consultation with the Stockholm University legal department. Requests for data, stored at the Stress Research Institute, Department of Psychology, should be sent to registrator@su.se with reference to “The implication of physically demanding and hazardous work on retirement timing” or directly to the corresponding author.

## Supplementary Materials

The following supporting information can be downloaded at: https://www.mdpi.com/article/10.3390/ijerph19138123/s1, Table S1: Blue-collar workers. Interaction effect between binary age (64–76 vs. 59–63 years at baseline) and PDWT or PHWE on retired (vs. still in work); Table S2: White-collar workers. Interaction effect between binary age (64–76 vs. 59–63 years at baseline) and PDWT or PHWE on retired (vs. still in work); Table S3: Blue-collar workers. Exclusion of disability pension in Model 3 and 4. Outcome was retired (1) vs. still in work (0) two years later in relation to PDWT and PHWE in trichotomized variables; Table S4: Blue-collar workers. Self-rated general health and physical and mental abilities added as covariates. Outcome was retired (1) vs. still in work (0) two years later in relation to PDWT and PHWE in trichotomized variables; Table S5: White-collar workers. Self-rated general health and physical and mental abilities added as covariates. Outcome was retired (1) vs. still in work (0) two years later in relation to PDWT and PHWE in dichotomised variables.
