# Supplementary material for: The Implication of Physically Demanding and Hazardous Work on Retirement Timing
Source: Int J Environ Res Public Health. 2022 Jul 1;19(13):8123. doi: 10.3390/ijerph19138123 (PMC9265406; doi:10.3390/ijerph19138123)
Supplement: Supplementary file 1 [file ijerph-19-08123-s001.zip › ijerph-1776455-supplementary.pdf]

International Journal of
*Environmental Research and Public Health*

## Supplementary Materials

**Table S1.** Blue-collar workers. Interaction effect between binary age (64–76 vs. 59–63 years at baseline) and PDWT or PHWE on retired (vs. still in work).

| | Women OR (95% CI) | Men OR (95% CI) |
|---|---|---|
| **PDWT** | | |
| Main effect: | | |
| light (ref) | – | – |
| moderate | 1.18 (0.79; 1.78) | 0.85 (0.56; 1.29) |
| heavy | 1.82 (1.21; 2.74) * | 1.21 (0.83; 1.79) |
| Interaction with binary age (≥64): | – | – |
| moderate | 0.99 (0.39; 2.50) | 1.85 (0.87; 3.90) |
| heavy | 0.36 (0.14; 0.92) * | 1.71 (0.80; 3.65) |
| **PHWE** | | |
| Main effect: | | |
| low-level (ref) | – | – |
| moderate | 0.99 (0.69; 1.43) | 0.84 (0.52; 1.35) |
| high-level | 1.86 (1.06; 3.28) * | 0.99 (0.63; 1.55) |
| Interaction with binary age (≥64): | – | – |
| moderate | 1.55 (0.70; 3.42) | 2.57 (1.17; 5.63) * |
| high-level | 1.58 (0.30; 8.30) | 2.79 (1.28; 6.09) * |

Fully adjusted for age (linear, quadratic and binary age), wave (categorical), education, marital status, parental status, working time, and caring for a relative. * for $p < 0.05$.

**Table S2.** White-collar workers. Interaction effect between binary age (64–76 vs. 59–63 years at baseline) and PDWT or PHWE on retired (vs still in work).

| | Women OR (95% CI) | Men OR (95% CI) |
|---|---|---|
| **PDWT** | | |
| Main effect | 0.77 (0.62; 0.97) * | 0.71 (0.55; 0.92) * |
| Interaction with binary age (≥64) | 1.24 (0.83; 1.85) | 1.33 (0.85; 2.07) |
| **PHWE** | | |
| Main effect | 0.95 (0.77; 1.17) | 1.03 (0.80; 1.34) |
| Interaction with binary age (≥64) | 1.72 (1.15; 2.56) ** | 1.02 (0.64; 1.64) |

Fully adjusted for age (linear, quadratic and binary age), wave (categorical), education, marital status, parental status, working time, and caring for a relative. * for $p < 0.05$, ** for $p < 0.01$.

**Table S3.** Blue-collar workers. Exclusion of disability pension in Model 3 and 4**.** Outcome was retired (1) vs. still in work (0) two years later in relation to PDWT and PHWE in trichotomized variables.

| | Blue-Collar Women | | Blue-Collar Men | |
|---|---|---|---|---|
| | **Manuscript** | **Excl. Dis Pens** | **Manuscript** | **Excl. Dis Pens** |
| **Model 3:** | | | | |
| PDWT | | | | |
| light (ref) | – | – | – | – |
| moderate | 1.24 (0.86; 1.78) | 1.17 (0.81; 1.70) | 1.04 (0.74; 1.47) | 1.04 (0.73; 1.47) |
| heavy | 1.53 (1.07; 2.20) * | 1.49 (1.03; 2.16) * | 1.42 (1.02; 1.98) * | 1.48 (1.06; 2.08) * |
| PHWE | | | | |
| low-level (ref) | – | – | – | – |
| moderate | 1.08 (0.78; 1.49) | 1.09 (0.79; 1.52) | 1.20 (0.82; 1.76) | 1.30 (0.88; 1.92) |
| high-level | 2.01 (1.19; 3.41) * | 1.79 (1.04; 3.09) * | 1.42 (0.98; 2.06) † | 1.55 (1.06; 2.27) * |
| **Model 4:** | | | | |
| Interaction term PDWT and linear age: | | | | |
| age#light (ref) | – | – | – | – |
| moderate | 0.97 (0.74; 1.28) | 1.07 (0.80; 1.43) | 1.06 (0.87; 1.30) | 1.09 (0.88; 1.34) |
| high-level | 0.77 (0.61; 0.99) * | 0.81 (0.63; 1.03) † | 1.10 (0.90; 1.36) | 1.05 (0.85; 1.30) |
| Interaction term PHWE and linear age: | | | | |
| low-level (ref) | – | – | – | – |
| moderate | 1.02 (0.81; 1.28) | 1.01 (0.80; 1.28) | 1.21 (0.98; 1.51) † | 1.17 (0.93; 1.48) |
| high-level | 0.91 (0.66; 1.26) | 0.98 (0.69; 1.39) | 1.26 (1.02; 1.56) * | 1.21 (0.97; 1.50) † |

* for $p < 0.05$; † for $0.05 \leq p < 0.10$.

**Table S4.** Blue-collar workers. Self-rated general health and physical & mental abilities added as covariates. Outcome was retired (1) vs. still in work (0) two years later in relation to PDWT and PHWE in trichotomized variables.

| | Blue-Collar Women | | Blue-Collar Men | |
|---|---|---|---|---|
| | **Manuscript** | **Modified** | **Manuscript** | **Modified** |
| **Model 3:** | | | | |
| **PDWT** | | | | |
| light (ref) | - | - | - | - |
| moderate | 1.24 (0.86; 1.78) | 1.23 (0.85; 1.79) | 1.04 (0.74; 1.47) | 1.06 (0.75; 1.51) |
| heavy | 1.53 (1.07; 2.20) * | 1.53 (1.05; 2.22) * | 1.42 (1.02; 1.98) * | 1.45 (1.03; 2.05) * |
| **PHWE** | | | | |
| low-level (ref) | - | - | - | - |
| moderate | 1.08 (0.78; 1.49) | 1.06 (0.76; 1.49) | 1.20 (0.82; 1.76) | 1.11 (0.75; 1.64) |
| high-level | 2.01 (1.19; 3.41) * | 1.78 (1.04; 3.06) * | 1.42 (0.98; 2.06) † | 1.24 (0.85; 1.82) |
| **Model 4:** | | | | |
| Interaction term **PDWT** and linear age: | | | | |
| age#light (ref) | – | – | – | – |
| moderate | 0.97 (0.74; 1.28) | 0.97 (0.73; 1.28) | 1.06 (0.87; 1.30) | 1.04 (0.85; 1.28) |
| high-level | 0.77 (0.61; 0.99) * | 0.79 (0.61; 1.01) † | 1.10 (0.90; 1.36) | 1.06 (0.86; 1.31) |
| Interaction term **PHWE** and linear age: | | | | |
| age#low-level (ref) | – | – | – | – |
| moderate | 1.02 (0.81; 1.28) | 1.02 (0.81; 1.28) | 1.21 (0.98; 1.51) † | 1.21 (0.97; 1.51) † |
| high-level | 0.91 (0.66; 1.26) | 0.94 (0.67; 1.31) | 1.26 (1.02; 1.56) * | 1.26 (1.02; 1.55) * |

* for $p < 0.05$; † for $0.05 \leq p < 0.10$.

**Table S5.** White-collar workers. Self-rated general health and physical and mental abilities added as covariates**.** Outcome was retired (1) vs. still in work (0) two years later in relation to PDWT and PHWE in dichotomised variables.

| | White-Collar Women | | White-Collar Men | |
|---|---|---|---|---|
| | **Manuscript** | **Modified** | **Manuscript** | **Modified** |
| **Model 3:** | | | | |
| PDWT | 0.84 (0.70; 1.00) † | 0.82 (0.69; 0.99) * | 0.78 (0.63; 0.96) * | 0.74 (0.60; 0.92) ** |
| PHWE | 1.10 (0.92; 1.31) | 1.06 (0.89; 1.27) | 1.04 (0.84; 1.30) | 0.99 (0.79; 1.24) |
| **Model 4:** | | | | |
| Interaction term PDWT and linear age | 1.08 (0.97; 1.21) | 1.08 (0.97; 1.21) | 1.10 (0.98; 1.24) | 1.10 (0.97; 1.24) |
| Interaction term PHWE and linear age | 1.20 (1.08; 1.34) *** | 1.19 (1.07; 1.33) *** | 0.99 (0.87; 1.11) | 0.98 (0.86; 1.10) |

*** for $p < 0.001$; ** for $p < 0.01$; * for $p < 0.05$; † for $0.05 \le p < 0.10$.
